# Fisetin-induced cell death in human ovarian cancer cell lines via zbp1-mediated necroptosis

**DOI:** 10.1186/s13048-022-00984-4

**Published:** 2022-05-10

**Authors:** Yaxian Liu, Hongwen Cao, Yanhui Zhao, Lijuan Shan, Shuhai Lan

**Affiliations:** Department of Obstetrics and Gynecology, Baodi Clinical College of Tianjin Medical University, Tianjin, 301800 China

**Keywords:** Gynecological malignancy, Cancer, Fisetin, Necroptosis

## Abstract

**Background:**

Among reproductive cancers, ovarian cancer leads to the highest female mortality rate. Fisetin, a natural flavonoid, exerts pharmacological effects, inhibiting cancer growth with various origins. Although multiple mechanisms are involved in regulating cell death, it is still unclear whether and how fisetin exhibits anticancer effects on ovarian cancer. The present study aimed to evaluate cell apoptotic and necroptotic processes occurring in ovarian carcinoma (OC) cell lines induced by fisetin.

**Methods:**

Cell growth was evaluated by MTT assay in OC cell lines treated with or without fisetin. Annexin V/propidium iodide staining followed by flow cytometry was used to characterize fisetin-induced cell death. The apoptotic process was suppressed by z-VAD intervention, and cell necroptosis was assessed by introducing ZBP1-knockdown OC cell lines coupled with fisetin intervention. The expression of necroptosis-related mediators and the migration capability of the respective cells were evaluated by Western blotting and in vitro cell invasion assay.

**Result:**

Fisetin successfully reduced cell growth in both OC cell lines in a dose-dependent manner. Both apoptosis and necroptosis were induced by fisetin. Suppression of the cell apoptotic process failed to enhance the proliferation of fisetin-treated cells. The induced cell death and robust expression of the necroptotic markers RIP3 and MLKL were alleviated by knocking down the expression of the ZBP1 protein in both OC cell lines.

**Conclusion:**

The present study provided in vitro evidence supporting the involvement of both apoptosis and necroptosis in fisetin-induced OC cell death, while ZBP1 regulates the necroptotic process via the RIP3/MLKL pathway.

## Introduction

Ovarian cancer (OC) ranks as the second most common gynecological malignancy and is also one of the leading causes of cancer-related mortality in women [1]. Although multiple types of chemotherapies and surgeries have been applied in OC treatment, the prognosis and long-term survival rate of OC patients are still poor [2, 3]. The high heterogeneity and complex cellular origins of OC still challenge the abilities of researchers and clinicians to screen substances and target critical factors that are able to effectively promote cell death or control ovarian carcinogenesis [4]. However, OC cells depend on both inherent and acquired mechanisms to resist the effect of chemotherapeutics and escape cell death [5]. Therefore, understanding the process and mechanisms involved in OC cell death under chemotherapies has become important for optimizing the current treatment outcome of ovarian cancer.

Fisetin (7,3’,4’-flavon-3-ol) is a flavonoid substance that can be extracted from a variety of plants, such as cucumbers, onions, apples and strawberries [6]. Fisetin has been reported to participate in multiple biological processes with therapeutic effects, including antioxidant effects, anti-inflammatory effects, and prevention or inhibition of cancer development [7–10]. As for its anticancer effects, many types of malignant cancer cells are affected. Fisetin arrested the cell cycle in colon cancer cells by inhibiting the activities of cyclin-dependent kinases [11]. In osteosarcoma, fisetin promotes cell death via mitochondrial apoptosis, cell cycle arrest and inhibition of cell migration [12] or by mediating multiple signaling pathways[13]. Jia et al. found that fisetin reduced the proliferation of pancreatic cancer cells by inducing autophagy via endoplasmic reticulum- and mitochondrion-related pathways [14].

Similar to those in other solid malignant cancers, various types of cell death processes have been discovered in OC over the past decade. Apoptosis occurs via two main mechanisms: a receptor-dependent extrinsic pathway and a mitochondrion-dependent intrinsic pathway [15]. Both pathways have been reported to lead to ovarian cancer cell death [16–18]. However, ovarian cancer cells are also able to escape therapeutic-induced apoptosis by counterbalancing the effectors and antagonists regulating caspase-related pathways [19, 20]. Necroptosis serves as an alternative approach to programmed cell death in a caspase-independent pathway and participates in the regulation of cancer metastasis, tumor progression or cancer immunity, which may be another significant mechanism for optimizing ovarian cancer treatment outcomes [21, 22]. Ferroptosis and the tumor microenvironment also participate in initiating OC cell death [23, 24].

In this study, we focused on necroptosis of OC via fisetin intervention. Recent studies have identified ZBP1 as a potential mediator in the regulation of cell death, including that in various types of cancer [25]. However, there is still a lack of evidence for how ZBP1 regulates cell death in ovarian cancer. To gain insight into the mechanism of fisetin in the treatment of ovarian cancer, in this study, we first proved that the cell death of different OC cell lines, A2780 and VOCAR-3, can be initiated by fisetin in a dose-dependent manner. Then, both cell apoptosis and necroptosis were shown to be involved in cell death induced by fisetin. After applying the inhibiting apoptotic approach, we observed that ZBP1 played a significant role in mediating fisetin-induced cell necroptosis in OC cell lines via the ZBP1-mediated RIP3/MLKL necroptotic pathway.

## Materials and Methods

### Reagents and antibodies

Fisetin and z-VAD were purchased from Sigma, USA. The primary antibodies used in this study for Western blotting were as follows: anti-HMGB1 (Invitrogen, NY, USA), 1:1000 dilution; anti-ZBP1 (R&D system, USA), 1:1000 dilution; anti-RIP3 (Calbiochem, San Diego, CA), 1:1000 dilution; anti-RIP1 (Calbiochem, San Diego, CA), 1:1000 dilution; anti-MLKL (BD Biosciences, USA), 1:1000 dilution; and anti-GAPDH (Santa Cruz, CA, USA), 1:5000 dilution.

### Cell culture

Human ovarian carcinoma (OC) cell lines were obtained from Procell (Procell, China) and cultured in DMEM (HyClone, USA) supplemented with 10% fetal bovine serum (Gibco, USA) and 1% penicillin–streptomycin (Sigma, USA). Both cell lines were cultured in a 37% with 5% CO_2_ atmosphere. Fisetin was dissolved in dimethyl sulfoxide (DMSO) to a series of concentrations of 0, 25, 50 or 100 µmol/L and then added to the culture medium to treat both cell lines for 72 h. v-ZAD (dissolved at a concentration of 30 μmol/L) was added to 100 µmol/L fisetin-treated cells for 72 h to inhibit the apoptotic process.

### Cell Proliferation Assay

The effect of fisetin on cell proliferation was examined by 3-(4,5- dimethylthiazol-2-yl)-2,5-diphenyl-2H-tetrazolium bromide (MTT) assay. Briefly, A2780 and VOCAR-3 cells in both the treated and control groups were seeded in 96-well plates at a concentration of 75 µmol/L cells per well. Cells were then incubated for 12 h following fisetin treatment at the series of concentrations mentioned above. After incubation for 72 h, MTT was added to each well, followed by 4 h of incubation. The medium was then washed 3 times to remove MTT. After stabilizing the blue formazan crystals by adding 150 µL of DMSO, the absorbance at 570 nm was detected with a microplate reader (Bio-Tek Instruments, VT) to determine the concentration of blue crystals.

### Cell death analysis

An Annexin V-FITC apoptosis detection kit (Sigma–Aldrich) was used for the detection of apoptotic cells. Cells were double-stained with PI and Annexin-V with binding buffer for 30 min at room temperature (RT). Apoptotic and necroptotic cells were analyzed with flow cytometry.

### Transfection of ZBP1 siRNA

ZBP1 siRNAs (Santa Cruz Biotechnology, Santa Cruz, CA) were introduced to block endogenous ZBP1 gene expression. Briefly, ZBP1 siRNA (Thermo Fisher, USA) was transfected into A2780 cells using an siRNA transfection kit (Santa Cruz Biotechnology, Santa Cruz, CA) following the manufacturer’s instructions. After 24 h of transfection, fisetin + z-VAD treatment was followed. The levels of ZBP1 protein in both cell lines were analyzed by Western blotting.

### In vitro invasion assay

An in vitro cell invasion assay was performed to measure the migration ability of both ZBP-knockdown (ZBP1-/-) and control cells after fisetin + z-VAD treatment using a Matrigel-coated Transwell chamber (Corning, MA, United States). Briefly, both types of cells at a density of 24 cells/well were seeded into the upper chambers in serum-free medium with fisetin + z-VAD, while DMEM containing 10% FBS and 1% penicillin–streptomycin was added to the lower chamber. After incubation for 24 h, the cells on the top surface were removed, while the cells on the lower surface of the membranes were fixed with 4% paraformaldehyde for 10 min and stained using 0.25% crystal violet for 15 min. The invaded cells were subjected to an MTT assay.

### Cell mitochondrion isolation

Cell mitochondrion isolation was performed using a Cell Mitochondria Isolation Kit (Thermal Fisher, USA) according to the manufacturer’s instructions. Briefly, the cells were washed with PBS and digested with trypsin, and then the cells were centrifuged at a speed of 100–200 × g for 5 min to collect alkalotic cells. We then transferred the cells to clean tubes and homogenized the cells 10–30 times. The cell homogenate was centrifuged at a speed of 600 g at 4 °C for 10 min. We transferred the supernatant to another clean tube and centrifuged it at a speed of 11,000 rpm at 4 °C for 10 min. The supernatant was cytosol, and the deposit was mitochondria.

### Real-time PCR

RNA was extracted from isolated mitochondria by the PureLink RNA mini kit (Thermo Fisher Scientific, USA). The quantity and quality of extracted RNA were analyzed by using a NanoDrop 2000 spectrophotometer. Extracted RNA was prepared to synthesize cDNA according to the QuantiNova Reverse Transcription Kit. The QuantiNova SYBR Green PCR Kit was used to determine mRNA expression on an Applied Biosystems PCR machine (95 °C for 10 min, 40 cycles of 95 °C for 15 s and 60 °C for 30 s, and a final extension step of 60 °C for 30 s). The sequences of the primers used in the qRT–PCR experiment were as follows:

Cyt-c forward primer, 5’-CTG GGTGACGAGTGAAACTG-3’; reverse primer, 5’-TGAGCACAACAGGAACTGGA-3’; primer length in bp, 104 bp;

GAPDH forward primer, 5’-GAAATCCCATCACCATCTTCCAGG-3’; reverse primer, 5’-GAGCCCCAGCCTTCTCCATG-3’; primer length in bp, 120 bp.

### Western blot analysis

Cells were cultured in the absence or presence of the respective intervention and then harvested and lysed in RIPA buffer to collect total protein. After ice incubation for 30 min, lysates were centrifuged at 12,000 × g for 5 min to separate the supernatants. The concentration of protein contained in the supernatants was measured by a BCA kit according to the manufacturer’s instructions. Next, 30 µg denatured protein samples were separated on SDS–PAGE gels and then transferred to PVDF membranes (Bio–Rad). After incubation of the membranes with primary antibodies at 4 °C overnight, the samples were incubated with the secondary antibodies conjugated with horseradish peroxidase for 1 h at room temperature. The blots were visualized using a Leica Imaging System.

### Statistical Analysis

Statistical analysis was completed with SPSS 23.0 (SPSS Inc.; Chicago, IL, USA) and GraphPad Prism 6 (San Diego, CA) software. Data are presented as the mean ± standard deviation. Student’s t test was used to compare the means of groups, and P values ≤ 0.05 were considered significant.

## Results

Fisetin suppressed cell proliferation in human ovarian cancer cell lines in vitro.

**Fig. 1:**
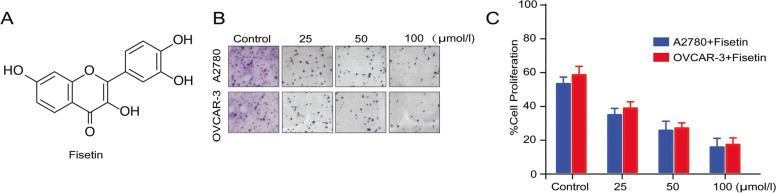
Fisetin inhibited the proliferation of both human ovary carcinoma cell lines (A2780 and OVCAR-3) in a dose-dependent manner. (A) The chemical structure of fisetin. (B) The MTT assay indicated that fisetin reduced the proliferation of both OC cell lines after treatment at incremental concentrations (25, 50 and 100 mmol/L) for 72 h. (C) Quantification of vital cells after fisetin treatment

To assess the cytotoxicity of fisetin (the chemical structure is shown in Fig. 1A) to OC cells, A2780 and VOCAR-3 cell lines were treated with fisetin at different concentrations (0, 25, 50 or 100 µmol/L) for 72 h to determine cell proliferation. The MTT assay indicated significant inhibition of cell proliferative activities in both cell lines after fisetin treatment at all concentrations (Fig. 1B). Only approximately 20% of cells of both cell lines survived after treatment with 100 µmol/L fisetin (Fig. 1C).

Fisetin not only induces apoptosis in OC cell lines.

**Fig. 2:**
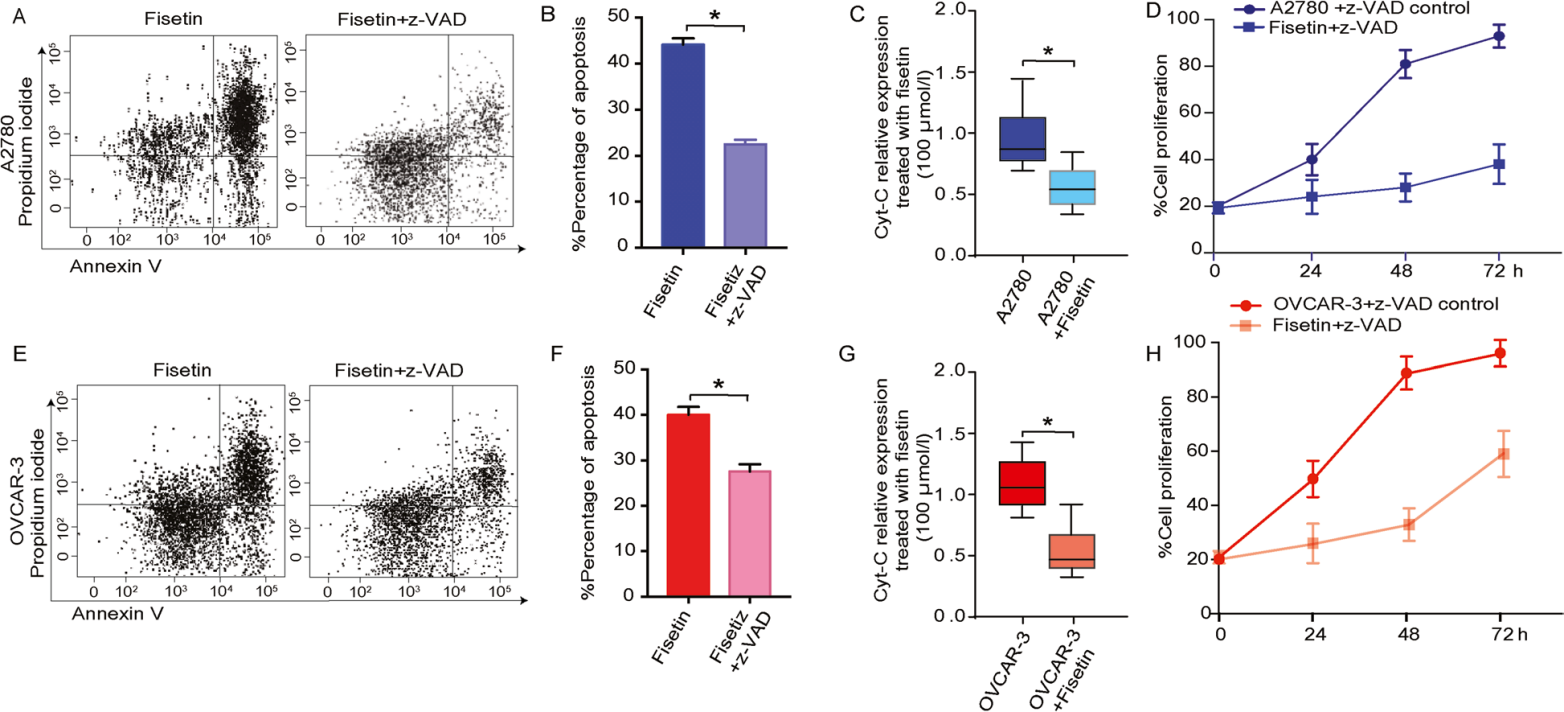
Mechanisms other than cell apoptosis involved in fisetin-induced OC cell death. (A) Cytometric results of annexin V and propidium iodide (AV/PI) staining of A2780 cells. (B) Quantification of apoptotic A2780 cells treated with fisetin ± z-VAD. (C) Relative expression level of Cyt-C translated in the mitochondria of A2780 cells treated with or without fisetin (100 mmol/L) for 72 h. (D) Comparison of fisetin-treated A2780 cell growth in a time-dependent manner, revealing that the inhibition of apoptosis by z-VAD failed to promote cell growth after fisetin treatment. (E–H) Identical experiments and intervention were performed for the other OC cell line, OVCAR-3. *, *p* < 0.05

The decrease in the number of vital cells might be attributed to robust cell death. Therefore, we verified our hypothesis by using Annexin V/propidium iodide (AV/PI) staining followed by flow cytometry analysis. Fisetin-treated A2780 cells showed a lower percentage of vital cells, as indicated by AV(-)/PI(-) (Fig. 2A). With intervention with z-VAD, a pan caspase inhibitor, fisetin-induced cell death was significantly reduced compared to that in cells without z-VAD treatment (Fig. 2B).

Due to the release of Cyt-c during apoptosis from mitochondria, the translation level of cyt-c mitochondrial RNA was significantly lower in fisetin-treated cells than in untreated cells in the present study, indicating that more intensive cell apoptosis was induced by fisetin (Fig. 2C). By inhibiting the apoptotic process using z-VAD, the percentage of apoptotic A2780 cells following fisetin treatment significantly decreased compared to that of cells without z-VAD antagonism. However, compared to the nontreated control group, suppression of A2780 cell apoptosis did not significantly enhance cell growth at different time points following fisetin treatment (Fig. 2D). Similar trends were also observed in the VOCAR-3 cell line (Fig. 2E-H). Consequently, the death induced by fisetin in OC cell lines is not simply attributed to apoptosis.

Fisetin-induced cell necroptosis was mediated by ZBP1 and involved the RIP3/MLKL pathway.

**Fig. 3:**
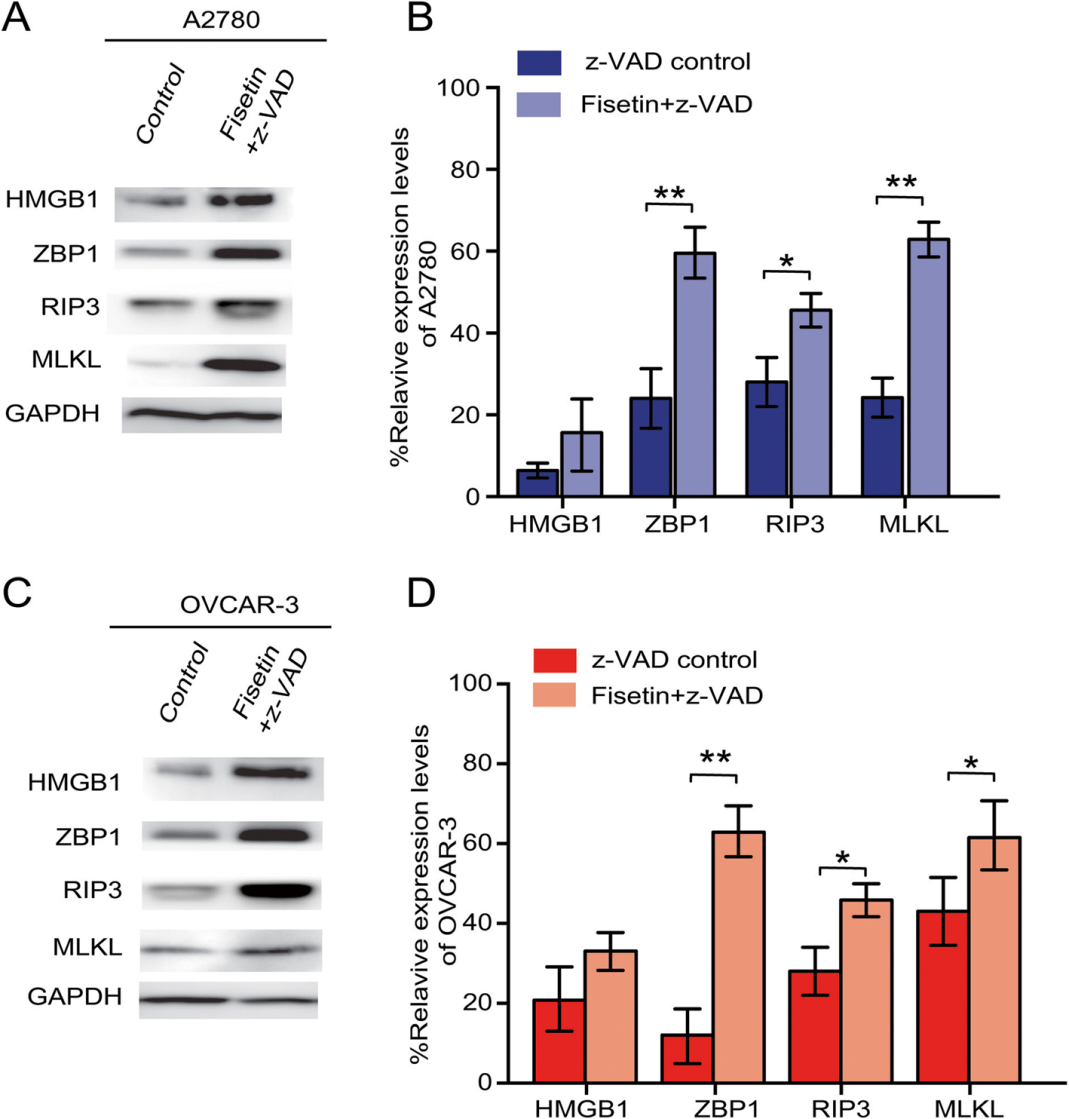
Cell necroptotic pathways regulate fisetin-induced cell death. (A) In A2780 cells, the expression of HMGB1, ZBP1, RIP3 and MLKL in the control and fisetin + z-VAD treatment groups was measured by Western blot. (B) Quantification and comparison of the levels of HMGB1, ZBP1, RIP3 and MLKL expression. (C, D) Western blot results and quantification of HMGB1, ZBP1, RIP3 and MLKL in OVCAR-3 cells under the respective interventions. *,* p* < 0.05

The expression of necroptotic markers was analyzed by Western blotting. A2780 cells treated with fisetin + z-VAD showed a significant increase in the protein levels of ZBP1, RIP3, and MLKL compared to those in the control, whereas the level of HMGB1, an inflammation-related marker, showed no significant difference between the groups (Fig. 3A-B). For the VOCAR-3 cell line, similar changes occurred between the fisetin and z-VAD groups compared to the nontreated control group (Fig. 3C-D). These results indicated that the necroptosis induced by fisetin in both OC cell lines was mediated by the ZBP1/RIP3/MLKL pathway.

ZBP1 knockdown compensated for the effects of fisetin + z-VAD intervention and restored the viability of OC cell lines.

**Fig. 4:**
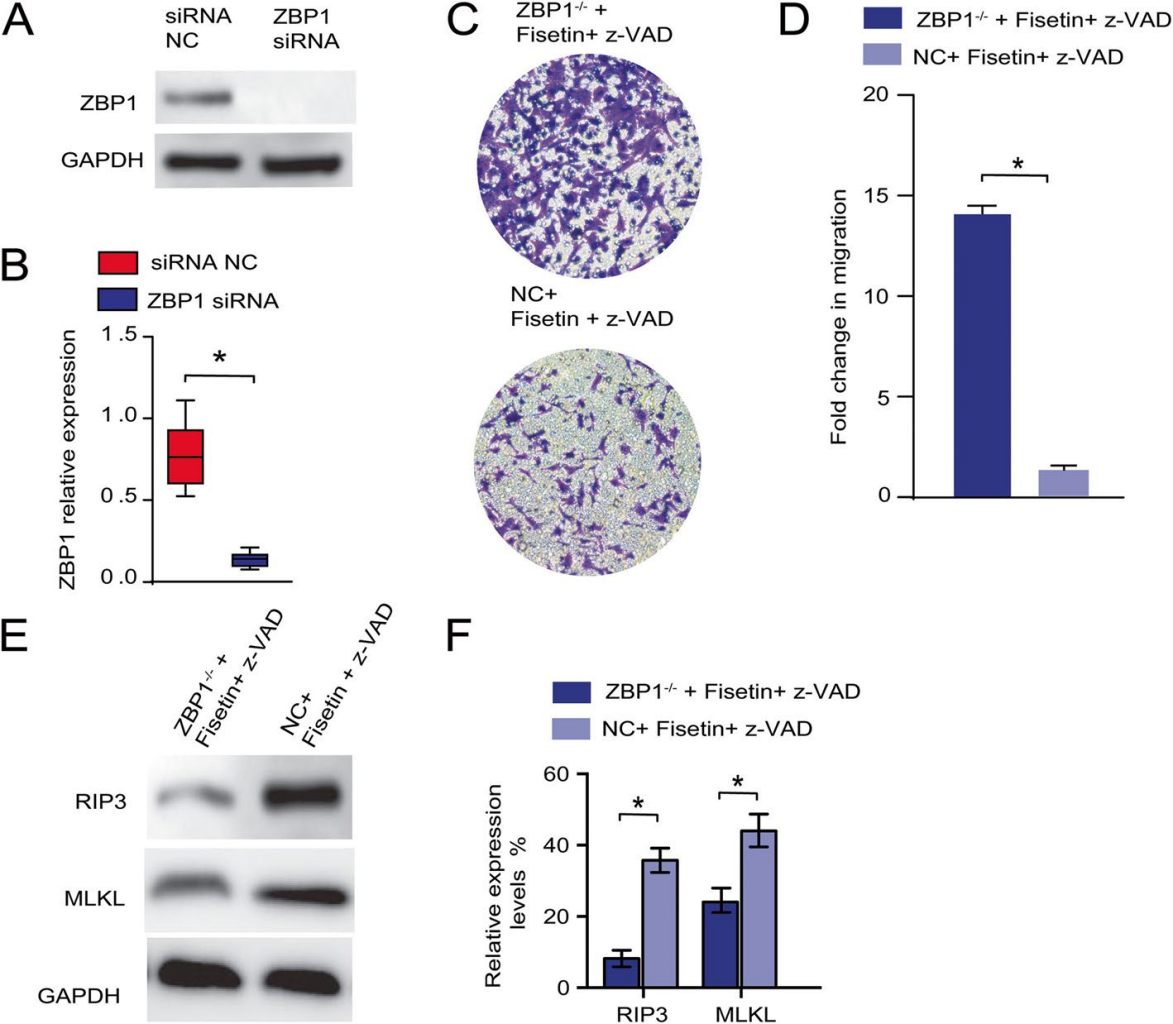
ZBP1 knockdown increased the migration of A2780 cells, blocking fisetin-induced apoptosis. (A) Western blotting of both the ZBP1 knockdown (ZBP1^−/−^) group and the control group, in which A2780 cells were treated with fisetin + z-VAD. (B) Quantification of ZBP1 expression with or without ZBP siRNA administration. (C) In vitro invasion assay between the respective groups. (D) MTT assay indicated differences in cell migration between the indicated groups. (E) Western blotting and (F) quantitative expression levels targeting RIP3 and MLKL in both ZBP1^−/−^ and normal A2780 cells, both of which were treated with fisetin + z-VAD. *, *p* < 0.05

To gain insights into the mechanism, we investigated how fisetin-induced OC cell necroptosis was regulated. Due to the possible dominant roles of the ZBP1/RIP3/MLKL pathway in controlling the necroptotic process, we hypothesized that ZBP1 plays a central role in mediating OC cell necroptosis following fisetin treatment. To eliminate the effects of the ZBP1 protein, A2780 cells were first transfected with siRNA interfering with ZBP1 expression. By Western blotting the ZBP1 level in either transfected or control cells, less ZBP1 protein was synthesized in ZBP1-/- cells than in control cells (Fig. 4A-B). Using these transfected cells in an in vitro invasion assay, we observed that the knockdown of ZBP1 blocked the necroptotic process in A2780 cells (Fig. 4C), as evidenced by the more robust migration activity of ZBP1-/- cells following fisetin + z-VAD treatment (Fig. 4D), whereas the presence of ZBP1 significantly inhibited cell migration in the control group (Fig. 4C, D). Next, we conjectured that a decline in endogenous ZBP1 levels might also impact cell necroptosis by downregulating the downstream markers RIP3 and MLKL. Western blot analysis of both markers showed significant inhibition in expression after ZBP1 was eliminated compared to that of the control group, in which OC cell necroptosis was induced by fisetin + z-VAD treatment (Fig. 4E). The relative expression level of downstream RIP3 was nearly 4 times lower than that in the nontransfected control (Fig. 4F). Similar inhibition was also observed for MLKL expression in fisetin + v-ZAD-induced cellular necroptosis (Fig. 4E-F).

**Fig. 5:**
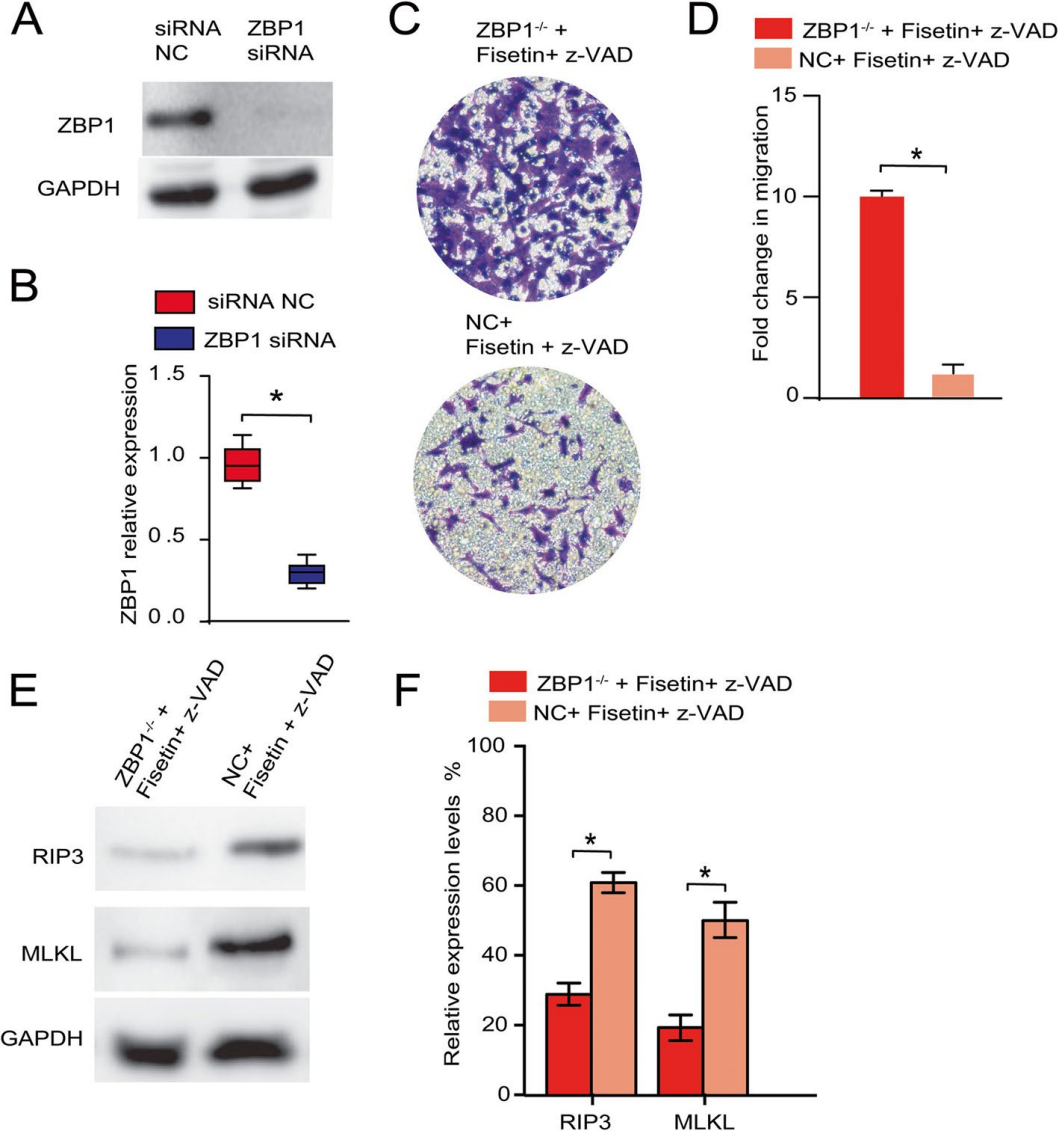
ZBP1 knockdown increased the migration of OVCAR-3 cells, blocking fisetin-induced apoptosis. **A** Western blotting of both the ZBP1 knockdown (ZBP1^−/−^) group and the control group, in which OVCAR-3 cells were treated with fisetin + z-VAD. (**B**) Quantification of ZBP1 expression with or without ZBP siRNA administration. (**C**) In vitro invasion assay between the respective groups. (**D**) MTT assay indicated differences in cell migration between the indicated groups. (**E**) Western blotting and (**F**) quantitative expression levels targeting RIP3 and MLKL in both ZBP1^−/−^ and normal OVCAR-3 cells, both of which were treated with fisetin + z-VAD. *, *p* < 0.05

ZBP1 siRNA also successfully blocked the targeted gene expression in OVCAR-3 cells (Fig. 5A, B), significantly compensating for the decreased cell migration and cell death (Fig. 5C, D). The RIP3 and MLKL expression levels showed similar suppression trends in OVCAR-3 cells with or without transfection (Fig. 5E, F), which were similar to those of the A2780 cell line. Consequently, we verified the initial hypothesis that ZBP1 acted as a central regulator of cell necroptosis in both the A2780 and OVCAR-3 cell lines.

## Discussion

In recent decades, humans have witnessed natural products becoming a new frontier in therapeutic development. Fisetin, a natural flavonoid, exerts pharmacological effects, mediating inflammation, inhibiting cancer, acting as an antioxidant and promoting angiogenesis [7–9]. It has been demonstrated as a phytochemical that exhibits cytotoxicity and inhibits proliferation in various types of cancer, including osteosarcoma [26], non-small cell lung carcinoma [27] and breast cancer [28]. These anticancer effects were also mediated by multiple signaling pathways related to cell death regulation. However, systemic research characterizing the properties and mechanism of fisetin in inhibiting ovarian cancer growth is still needed. The present study provides in vitro evidence demonstrating the effects of fisetin in promoting the death of various ovarian cancer cell lines via cell apoptosis and necroptosis. ZBP1 plays a key role in mediating the necroptotic process induced by fisetin in OC cell lines.

First, we introduced two types of cell lines representing human OC cells and verified the cytotoxicity of fisetin at different concentrations by MTT assay. The results indicated that fisetin inhibited the proliferation of both cell lines in a dose-dependent manner. Among all concentrations selected, 100 µmol/L showed the strongest inhibitory effects in our study. This dose-dependent effect of fisetin was verified in a similar study targeting the other type of OC cell line, namely, the SKOV3 cell line, in which up to 300 µmol/L fisetin was involved [29]. The results of the MTT assay demonstrated greater inhibition of cell viability in the 100 µmol/L group than in the 30 µmol/L group. Although it showed a more robust growth inhibitory effect in the 300 µmol/L group, the difference in cell viability between the 100 and 300 µmol/L groups was relatively small compared to that between the 30 and 100 µmol/L groups. Thus, 100 µmol/L fisetin was able to induce strong cell death in OC cell lines for further experiments.

Cell apoptosis is a common process involved in chemically induced cell death among various cancers [30, 31]. Studies have also reported that fisetin induces cell apoptosis in different types of malignant tumor cell lines. Here, we first verified this hypothesis by performing annexin V (AV) and propidium iodide (PI) double staining followed by flow cytometry analysis [32]. A higher percentage of AV( +)/PI( +) and AV(-)/PI( +) indicated that apoptosis at different stages was induced by fisetin, causing less expression of Cyt-c in fisetin-treated cells. Other studies demonstrated that fisetin induces apoptotic cell death through endoplasmic reticulum stress and the mitochondrion-dependent apoptotic pathway in human melanoma and oral cancer cells [33]. Apoptosis is morphologically, chemically, and pathophysiologically distinct from necrosis. The specific morphological changes of apoptosis are nuclear condensation, cell volume reduction and DNA fragmentation [34]. z-VAD acts as an effective pancaspase inhibitor that can significantly block the mitochondrion-driven intrinsic apoptotic process. However, compared to fisetin treatment alone, z-VAD administration was unable to reverse the inhibitory effects of fisetin on cell proliferation. These results indicated the possibility that mechanisms other than cell apoptosis might contribute to fisetin-induced cell death in the selected OC cell lines.

Meanwhile, the present cytometric results also indicated the possibility that cell necroptosis occurred, as evidenced by the strong AV( +)/PI( +) signal. Thus, we hypothesized that the cell necroptotic process and related signaling pathway regulate fisetin-induced OC cell death.

**Fig. 6:**
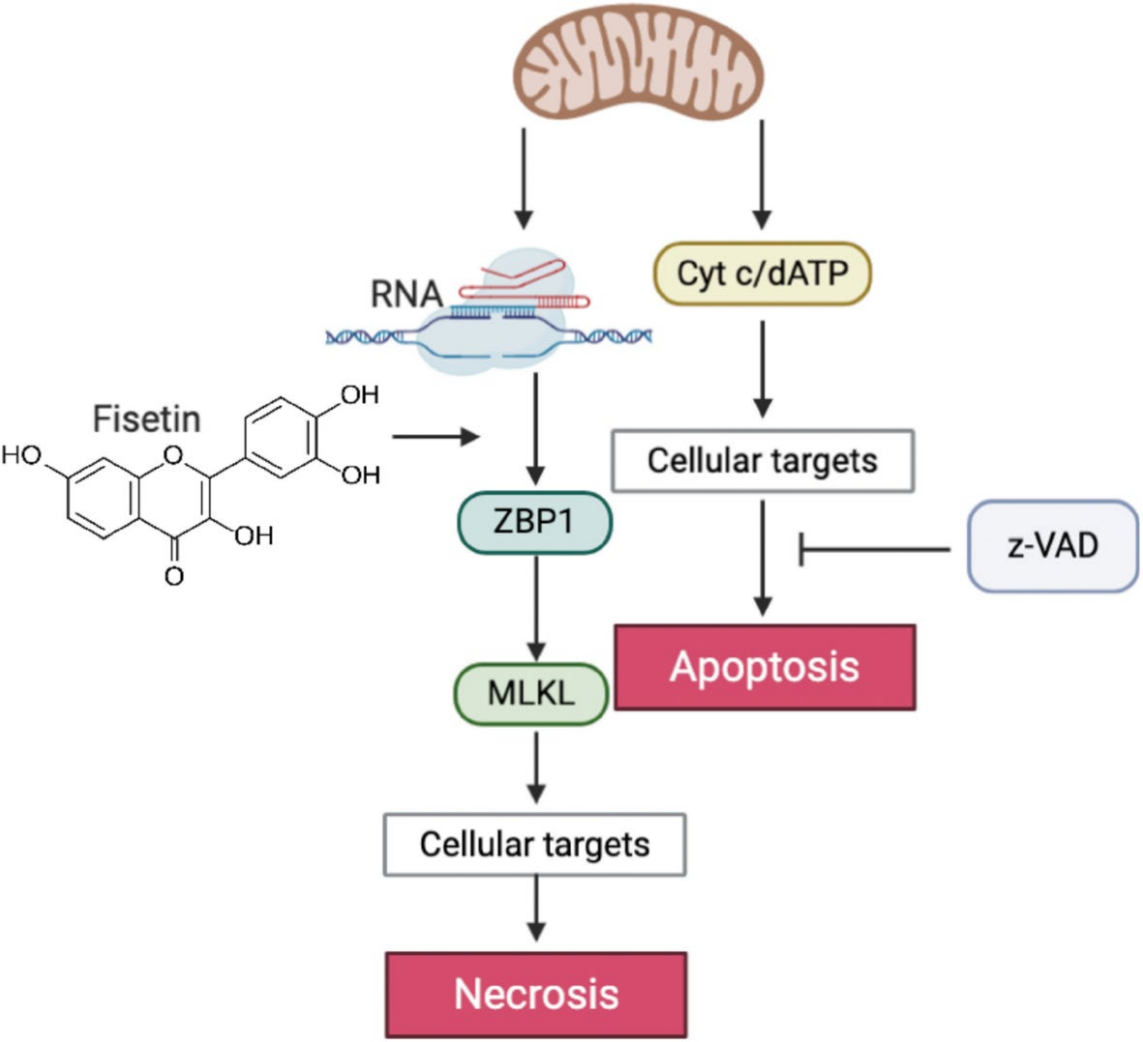
Schematic illustration of the mechanism underlying OC cell death induced by fisetin

Z-DNA-binding protein 1 (ZBP1) functions as a central regulator of cell necroptosis under diverse conditions [25, 35, 36]. ZBP1 facilitated RIPK3 activation and its binding to MLKL [36, 37]. The activation of RIP3 is key to activating MLKL-mediated cell necroptosis. In the present study, Western blotting targeting the expression levels of ZBP1, RIP3 and MLKL was performed in the OC cell lines with or without fisetin + z-VAD treatment. The results supported the hypothesis that cell necroptosis was attributable to cell death induced by fisetin. To validate the initiative role of ZBP1 in mediating necroptosis, we successfully established ZBP1 siRNA-transfected A2780 and OVCAR-3 cells to knock down ZBP1 expression. Then, the invasion assay demonstrated stronger migration in ZBP1-knockdown cells after fisetin + z-VAD treatment. The downstream mediators RIP3 and MLKL were also downregulated in the absence of ZBP1 (Fig. 6).

## Conclusions

To the best of our knowledge, the data presented in this study provide the first in vitro evidence that both cell apoptosis and necroptosis play important roles in the process of fisetin-initiated OC cell death. ZBP1 mediated fisetin-induced cell necroptosis in OC cell lines via the RIP3/MLKL pathway. However, the contribution of each mentioned mechanism to the cell death process by fisetin needs to be further quantified.

## Data Availability

The dataset supporting the conclusions of this article is included within the article and its additional files.
